# A novel model for expanding horizons in sign Language recognition

**DOI:** 10.1038/s41598-025-09643-2

**Published:** 2025-07-08

**Authors:** Esraa Hassan, Mahmoud Y. Shams, Tarek Abd El-Hafeez, Marwa Elseddik

**Affiliations:** 1 Department of Machine Learning and Information Retrieval, Faculty of Artificial Intelligence, Kafrelsheikh University, Kafr El-Sheikh, 33516 Egypt; 2 Computer Science Unit, Deraya University, El-Minia, Egypt; 3 Department of Computer Science, Faculty of Science, Minia University, El-Minia, Egypt; 4 Department of the Robotics and Intelligence Machines, Faculty of Artificial Intelligence, Kafrelsheikh University, Kafrelsheikh, 33516 Egypt

**Keywords:** Attention mechanism, Computer vision, American sign Language (ASL), Deep learning, Communication accessibility, Computer science, Information technology, Software, Health care, Signs and symptoms

## Abstract

The American Sign Language Recognition Dataset is a pivotal resource for research in visual-gestural languages for American Sign Language and Sign-Language MNIST Dataset. The dataset contains over 64,000 images meticulously labeled with the corresponding ASL gesture or letter to recognize and classify ASL signs. Recent computer vision and deep learning advances have enabled various ASL recognition techniques. However, further improvements in accuracy and robustness are still needed. This study comprehensively evaluates different ASL recognition methods and introduces a novel architecture called Sign Nevestro Densenet Attention (SNDA). All methods are evaluated on the ASL Recognition Dataset to ensure a representative evaluation. SNDA employs the Nadam optimizer for faster convergence during training. Accurate ASL classification has practical implications for improving communication accessibility. SNDA achieves state-of-the-art performance with 99.76% accuracy, perfect sensitivity, and high specificity and precision. These results validate the effectiveness of SNDA for ASL gesture recognition and highlight its potential to promote inclusivity for deaf and hard-of-hearing communities. The fused attention mechanism demonstrates how deep learning models can be enhanced for specific application domains.

## Introduction

American Sign Language (ASL) is a vital means of communication for the deaf and hard-of-hearing community^1^. The recent synergy between computer vision and machine learning technologies has paved the way for diverse ASL recognition techniques, aiming to amplify communication accessibility^2^. Despite notable progress, there persists a critical need for improvements in the accuracy and robustness of these recognition systems to bridge existing gaps by offering a comprehensive comparison of various ASL recognition techniques, benchmarked against the ASL Recognition Dataset. The dataset encompasses various ASL gestures captured under diverse lighting conditions and camera angles, ensuring a thorough evaluation of proposed methods^3^.

A groundbreaking ASL recognition architecture, Sign Nevestro Densenet Attention (SNDA), takes center stage. SNDA integrates advanced technologies, including DenseNet for efficient feature extraction and an attention mechanism focusing on crucial regions within ASL gestures. The architecture is optimized using the Nadam optimizer, ensuring faster convergence and stable optimization during model training^4–6^. The precise classification of ASL gestures holds profound implications for communication accessibility, positioning SNDA as a promising technology. Beyond ASL recognition, SNDA envisions contributions to ASL translation systems, assistive technologies, and educational tools, ushering in new avenues for inclusivity and empowerment within the deaf and hard-of-hearing community. The distinctive feature of SNDA lies in its fusion of attention mechanisms with established architectures to showcase the potential for enhancing deep learning models, particularly in ASL recognition.

### Problem statement

Despite breakthroughs in computer vision and machine learning, current ASL recognition techniques suffer from challenges in accuracy and robustness to impede effective communication for the deaf and hard-of-hearing community, limiting accessibility and social interaction. Existing methods encounter issues like misinterpreting ASL gestures, and sensitivity to variations in lighting and camera angles. This study addresses these challenges by proposing Sign Nevestro Densenet Attention (SNDA), a novel architecture designed explicitly for ASL recognition. SNDA aims to significantly improve recognition accuracy, enhance robustness to variations, and effectively.

### Contributions

1. Introducing the novel SNDA architecture, specifically designed for ASL recognition, incorporating DenseNet, attention mechanisms, and the Nadam optimizer.
2. Conducting a comprehensive evaluation of various ASL recognition techniques on the ASL Recognition Dataset, ensuring a representative assessment.
3. Illustrating SNDA’s ability to bridge communication gaps and foster.
4. Highlighting the benefits of combining attention mechanisms with established architectures, showcasing the potential for enhancing deep learning models in specific application domains.
5. DenseNet is chosen as the SNDA architecture’s foundation due to its feature reuse, gradient flow, rich representational power, and scalability. Its densely connected layers enable direct access to gradients, making it ideal for ASL recognition, distinguishing subtle variations in hand shapes and orientations.

The remainder of the paper unfolds as follows: Sect. 2 delves into the related work, Sect. 3 illustrates the proposed work, Sect. 4 delves into the results and discussion, and lastly, Sect. 5 presents the conclusion and outlines future avenues for research.

## Related work

Sign language recognition was a significant advancement in communication within the deaf-mute community and has been a focal point of research for years^7^. Previous studies had successfully recognized sign language, but they often required expensive equipment such as sensors, devices, and high-end processing power^8^. However, these limitations could be easily addressed by utilizing artificial intelligence-based techniques^9,10^. Artificial intelligence (AI) tools encompass various techniques and technologies, including convolutional neural networks (CNNs) for image recognition tasks^11–14^, recurrent neural networks (RNNs) like Long Short-Term Memory (LSTM) networks for capturing temporal dependencies in sign language sequences, transfer learning to accelerate model training and improve performance^15,16^, data augmentation techniques^17^ to enhance model robustness, gesture tracking and pose estimation algorithms to identify relevant hand gestures and spatial configurations, attention mechanisms for dynamically adjusting focus during inference, and multi-modal integration of depth information from 3D sensors or audio cues alongside visual signals to enhance accuracy and robustness. By leveraging these AI tools and techniques, researchers and developers aim to create more accurate, efficient, and accessible ASL recognition systems for the deaf and hard-of-hearing community. Speech impairment poses challenges for individuals in communicating via speech and hearing, leading them to rely on alternate means such as sign language^18^. While sign language is increasingly prevalent, there’s still a gap in communication between signers and non-signers^19^. Recent advancements in deep learning and computer vision offer promising solutions for motion and gesture recognition^20^.

Pan et al., 2016^21^ developed a vision-based gesture recognition system capable of handling complex backgrounds. Their method adjusted to varying skin colors and lighting conditions.

They merged three feature types: principal component analysis (PCA), linear discriminant analysis (LDA), and support vector machine (SVM) to characterize hand gestures. Their dataset encompassed 7800 images representing the ASL alphabet. The system achieved an accuracy of 94%.

Bantupalli and Xie, 2018^22^ proposed a model that can process video sequences, extracting both temporal and spatial features. Spatial features are recognized using Inception, a Convolutional Neural Network (CNN), while temporal features are trained using a Recurrent Neural Network (RNN). The American Sign Language Dataset serves as the dataset. In a related study, Morocho Cayamcela and Lim, 2019^23^ employed an identical dataset containing 87,000 images for classification purposes. They utilized the AlexNet and GoogLeNet models, achieving training accuracies of 99.39% for AlexNet and 95.52% for GoogLeNet.

Kasapbaşi et al., 2022^24^ presented a study that focused on creating a dataset and developing a Convolutional Neural Network (CNN)-based system for interpreting sign language gestures and hand poses into natural language. The CNN model, specifically tailored for the American Sign Language alphabet (ASLA), was designed to improve predictability. A new dataset for the ASLA was established, considering various conditions like lighting and distance, contributing to the field of sign language recognition (SLR). The dataset could potentially be utilized for further SLR system development. Additionally, the study compared the performance of its dataset with two others, noting that despite differences in conditions and dataset size, the proposed CNN model achieved a high accuracy of 99.38% with excellent prediction and minimal loss (0.0250).

Alsharif et al., 2023^3^ employed models such as AlexNet, ConvNeXt, EfficientNet, ResNet-50, and VisionTransformer, trained and tested on a dataset containing over 87,000 ASL alphabet hand gesture images. Various experiments were conducted to optimize model parameters for maximum accuracy. Results showed that ResNet-50 achieved the highest accuracy at 99.98%, followed by EfficientNet at 99.95%, ConvNeXt at 99.51%, AlexNet at 99.50%, and VisionTransformer at 88.59%. In this paper, we used a dataset that includes a diverse array of images meticulously labeled with corresponding ASL gestures or letters, this dataset supports supervised learning approaches for training models to recognize and classify ASL gestures effectively. With 1400 images for each alphabet, digits, and custom words like question marks and spaces, it covers a broad spectrum of linguistic expressions. The dataset is organized into training and test sets, each containing folders with identical names but differing images. The training set consists of 56,000 images, while the test set contains 8,000 images^25^. The proposed Sign Nevestro Densenet Attention (SNDA) algorithm achieved an impressive accuracy of 99.76%. Table 1 summarizes the recent efforts to classify and recognize the ASL and investigates the approach, methods, dataset, and results obtained.

**Table 1:** The recent efforts of ASL recognition compared with the proposed SNDA method.

Author	Approach	Method	Dataset	Results
Pan et al., 2016^21^	Vision-based	PCA, LDA, SVM	7800 ASL images	94% accuracy
Bantupalli & Xie, 2018^22^	Video sequences	Inception (CNN) + RNN	American Sign Language Dataset includes 2400 images, 1800 for training and 600 for testing	92% accuracy
Author	Approach	Method	Dataset	Results
Morocho Cayamcela & Lim, 2019^23^	Image classification	AlexNet & GoogLeNet	87,000 ASL images	99.39% (AlexNet), 95.52% (GoogLeNet)
Kasapbaşi et al., 2022^24^	CNN-based	Custom CNN for ASLA	New ASLA dataset	99.38% accuracy
Pathan et al., 2023^26^	Image-based	Multi-headed CNN	Finger Spelling, A dataset	98.98% accuracy
Alsharif et al., 2023^3^	Image classification	AlexNet, ConvNeXt, EfficientNet, ResNet-50, VisionTransformer	87,000 + ASL images	99.98% (ResNet-50), 99.95% (EfficientNet), 99.51% (ConvNeXt), 99.50% (AlexNet), 88.59% (VisionTransformer)

## The proposed work

In this section, we introduce a robust architecture for American Sign Language (ASL) called Sign Nevestro Densenet Attention (SNDA). This innovative approach offers several key contributions. SNDA enhances the accuracy of ASL gesture classification compared to conventional architectures. The attention mechanism within DenseNet enables the model to focus on the most informative parts of ASL signs, leading to more accurate and robust classifications^27^.

The DenseNet backbone^28^, characterized by its densely connected blocks, efficiently extracts intricate ASL gesture features. The concatenated feature maps across layers optimize feature propagation, enhancing the model’s ability to capture subtle variations in hand shapes, orientations, and movement dynamics^29^. Incorporating an attention mechanism dynamically highlights relevant regions within ASL gesture images during feature extraction. The attention-driven approach allows SNDA to adaptively emphasize informative parts of signs, improving model performance and robustness to variations in hand positioning and movements. SNDA employs the Nadam optimizer, an advanced variant of the Adam optimizer. The Nadam optimizer’s adaptive learning rate and momentum parameters facilitate faster convergence and more stable optimization during model training, resulting in more efficient and effective training processes^30^. Accurate classification of ASL gestures holds practical implications for improving communication accessibility for the Deaf and hard-of-hearing community. SNDA’s superior performance contributes to better ASL recognition systems, bridging communication gaps and fostering inclusivity. Its potential extends beyond research to positively impact society. By introducing SNDA, we contribute to the ongoing advancement of ASL recognition technology, pushing the boundaries of achievable accuracy and robustness in ASL gesture classification. SNDA represents a novel and innovative approach to ASL gesture recognition, showcasing the potential for combining attention mechanisms with established architectures. The innovative fusion of concepts contributes to the scientific understanding of how attention mechanisms can enhance deep learning models in specific application domains. The SNDA architecture is composed of the main components:

1. **Dense net backbone**: The foundation of SNDA is the DenseNet architecture. DenseNet’s unique densely connected blocks, as mathematically represented by Eq. (1) as follows:

1 $$\:{H}_{l}={H}_{l-1}+BN\left(ReLU\right(Conv\left({H}_{l-1}\right)\left)\right)$$

where *H*_*l*_ represents the feature maps at layer l, *Conv* denotes the convolution operation, *BN* signifies batch normalization, and *ReLU* represents the rectified linear unit activation function, fostering feature extraction prowess. DenseNet optimizes feature propagation by concatenating outputs across layers, enabling the model to capture convoluted ASL gesture features efficiently.

2. **Attention mechanism**: A valuable enhancement in SNDA is the integration of an attention mechanism within the Dense Net architecture. The attention mechanism is formally defined in Eq. (2).

2 $$\:A\left(x\right)=\sigma\:\left(softmax\right({W}_{2}\delta\:\left({W}_{1}X\right)\left)\right)$$

where X represents feature maps, *W*_*1*_ and *W*_*2*_ denote learnable weights, delta represents a non-linear activation function, and sigma denotes the sigmoid activation function. The attention mechanism dynamically highlights relevant regions within the ASL gesture images during feature extraction. It computes attention scores for each spatial location, enabling the model to focus on the most informative parts of ASL signs. Mathematically, the attention mechanism modulates the feature maps *X* through element-wise multiplication with the attention scores *A(X)*.

3. **Nadam optimizer**: Model training within the SNDA architecture is facilitated by the Nadam optimizer, an extension of the Adam optimizer. The Nadam optimizer adapts the learning rate and momentum during training, as governed by the following mathematical formulations Eqs. (3), (4), (5), (6), and (7).

3 $$\:{m}_{t}={\beta}_{1}{m}_{t-1}+(1-{\beta}_{1}){g}_{t}$$

4 $$\:{v}_{t}={\beta}_{2}{v}_{t-1}+(1-{\beta}_{2}){g}_{t}^{2}$$

5 $$\:{\widehat{m}}_{t}=\frac{{m}_{t}}{1-{\beta}_{1}^{t}}$$

6 $$\:{\widehat{v}}_{t}=\frac{{mv}_{t}}{1-{\beta}_{2}^{t}}$$

7 $$\:{w}_{t+1}={w}_{t}-\frac{\eta\:}{\sqrt{{\widehat{v}}_{t}}+\epsilon}\odot\:({\beta}_{1}{\widehat{m}}_{t}+(1-{\beta}_{1}\left){g}_{t}\right)$$

where $\:{m}_{t}$ and $\:{v}_{t}$ represent the first and second moments of gradients at time *t*, $\:{\beta\:}_{1}$ and $\:{\beta\:}_{2}$ are hyperparameters controlling exponential decay rates, $\:{and\:w}_{t}$ signifies the model parameters at time *t*. $\:\eta\:$ is the learning rate, $\epsilon$ prevents division by zero, and $\:\odot\:$ denotes element-wise multiplication. To ascertain the efficacy of the SNDA model, we compare its performance against established pre-trained architectures:

4. **ResNet**: Residual Networks, characterized by their depth and skip connections, are represented mathematically as in Eq. (8).

8 $$\:{H}_{l}=\:F({H}_{l-1},{W}_{l})+{H}_{l-1}$$

where *H*_*l*_ denotes the feature maps at layer l, *F* represents the residual mapping function, and $\:{W}_{l}$ is the layer-specific weights. ResNet’s architecture is designed to mitigate vanishing gradient problems and facilitates the training of very deep networks.

**Fig. 1:**
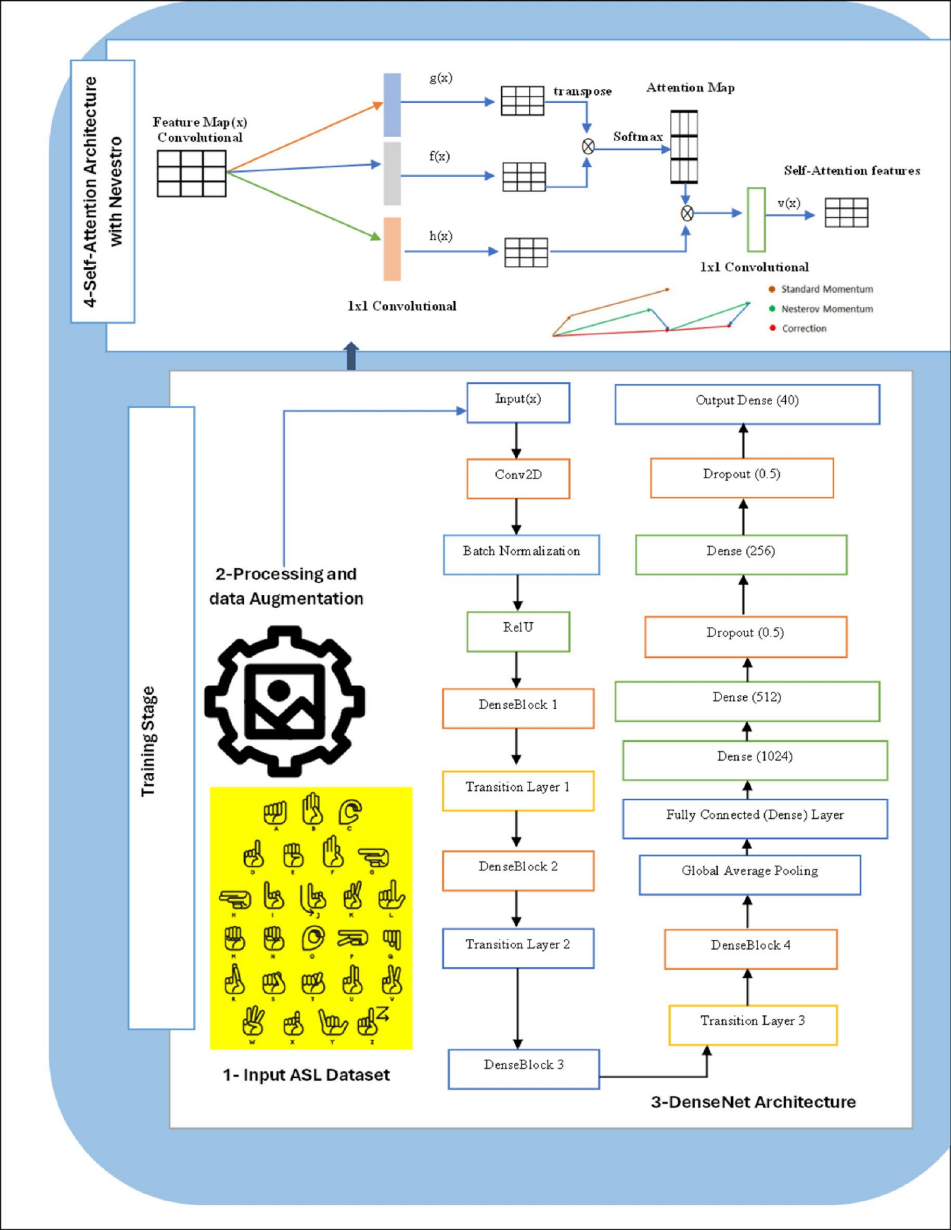
The architecture of the proposed SNDA model.

The ASL recognition process using the proposed SNDA model entails several key steps. Initially, a comprehensive ASL dataset comprising 1400 images for each alphabet, digit, and custom word is curated and organized into training and test sets. These images undergo preprocessing and data augmentation techniques to enhance quality and diversity. The DenseNet architecture serves as the backbone for feature extraction, efficiently capturing intricate ASL gesture features. Additionally, a self-attention mechanism with Nevestro dynamically highlights relevant regions within the images, improving model performance and robustness. Through the integration of these components, the SNDA model achieves superior accuracy in ASL gesture classification, contributing to the advancement of ASL recognition technology. Figure 1 illustrates the architecture of the proposed SNDA model, while Algorithms 1 delineate the steps of the proposed SNDA model.

The custom code and algorithms used to generate the results in this study are publicly available under an open-source license at: https://github.com/tarekhemdan/ASL. A versioned DOI (10.5281/zenodo.15725210) has been minted via Zenodo to ensure long-term accessibility. All test data required to replicate the findings are included in the repository (https://zenodo.org/records/15725210).

**Algorithm 1:**
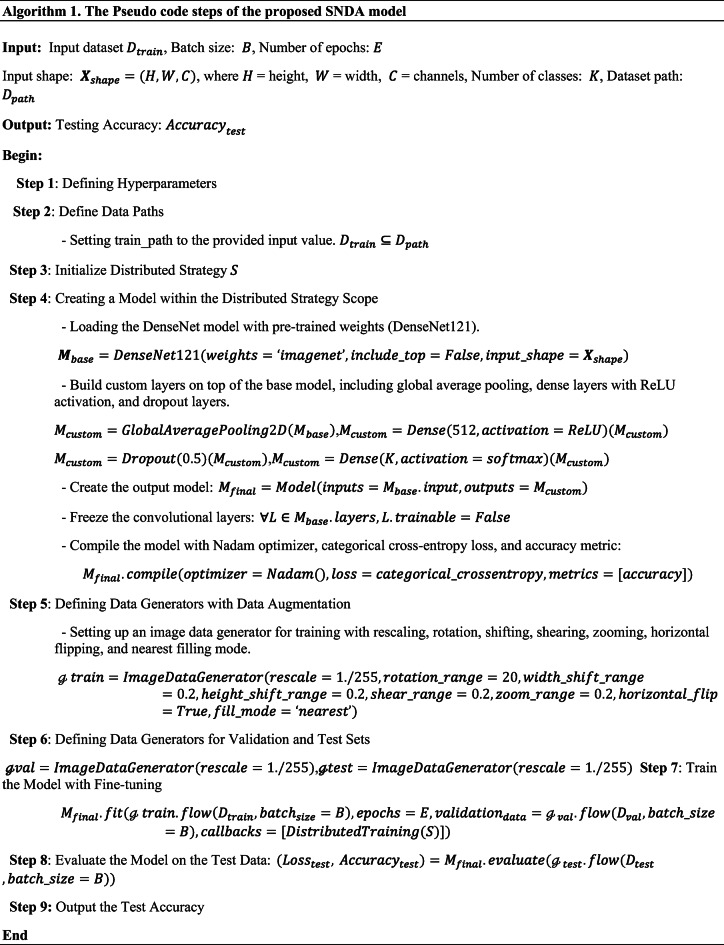
The steps of the proposed SNDA model.

## Results and discussion

### Datasets

#### ASL dataset

The American Sign Language Recognition Dataset serves as a pivotal resource in the realm of visual-gestural languages, particularly utilized by individuals with hearing impairments and within the deaf community. The dataset encapsulates the intricacies of American Sign Language (ASL) through hand and arm movements, paving the way for the development of cutting-edge machine learning algorithms and computer vision techniques. The primary goal is to foster accurate interpretation and translation of ASL gestures into textual or other communicative forms. Available on the Kaggle platform and curated by Rahul Makwana, this dataset has emerged as a foundational tool for advancing research and innovation in ASL recognition. The dataset boasts a rich diversity of images, each meticulously labeled with the corresponding ASL gesture or letter it signifies. This labeling strategy serves as a cornerstone for supervised learning approaches, enabling the training of models to proficiently recognize and classify ASL gestures based on the provided images. Comprising a total of 1400 images for each alphabet, digits, and custom words like question marks and spaces, the ASL dataset caters to a comprehensive range of linguistic expressions. Notably, the dataset is thoughtfully organized into two distinct folders: the training set and the test set. Although both sets share identical folder names, the images within the test set differ from those in the training set. The training set encompasses a substantial collection of 56,000 images, while the test set comprises 8,000 images^25^.

#### Sign-Language MNIST dataset

The Sign Language MNIST dataset is a challenging benchmark for image-based machine learning methods, focusing on American Sign Language hand gestures. It is relevant for assistive technology applications for the deaf and hard-of-hearing community.

### Evaluation metrics

In evaluating the performance of the proposed SNDA model, several key metrics are used to assess their effectiveness. Here, we define and provide equations for four commonly used metrics^31^:

1. **Accuracy** represents the proportion of correct predictions made by the model. It considers both true positives (correctly identified positive cases) and true negatives (correctly identified negative cases) as shown in Eq. (9)^32–34^.

9 $$\:Accuracy\:=\:\frac{{T}_{P}+{T}_{N}}{{T}_{P}+{T}_{N}+{F}_{P}+{F}_{N}}$$

where T_P_ is the true positives (correctly predicted positive cases), T_N_ is the true negatives (correctly predicted negative cases), F_P_ is the false positives (incorrectly predicted positive cases), and F_N_ is the false negatives (incorrectly predicted negative cases).

2. **Sensitivity**,** or recall**, measures the ability of the model to correctly identify positive cases. It expresses the proportion of actual positive cases that the model correctly predicted as positive as shown in Eq. (10)^35^.

10 $$\:Sensitivity\:=\:\frac{{T}_{P}}{{T}_{P}+{F}_{N}}$$

3. **Specificity** measures the ability of the model to correctly identify negative cases. It expresses the proportion of actual negative cases that the model correctly predicted as negative as shown in Eq. (11).

11 $$\:Specificity\:=\:\frac{{T}_{N}}{{T}_{N}+{F}_{P}}$$

4. **Precision** measures the proportion of correct positive predictions. It expresses the ratio of true positives to the total number of positive predictions made by the model as shown in Eq. (12)^36–38^.

12 $$\:Precision\:=\:\frac{{T}_{P}}{{T}_{P}+{F}_{P}}$$

### Experimental results

#### Statistical analysis for ASL dataset

**Fig. 2:**
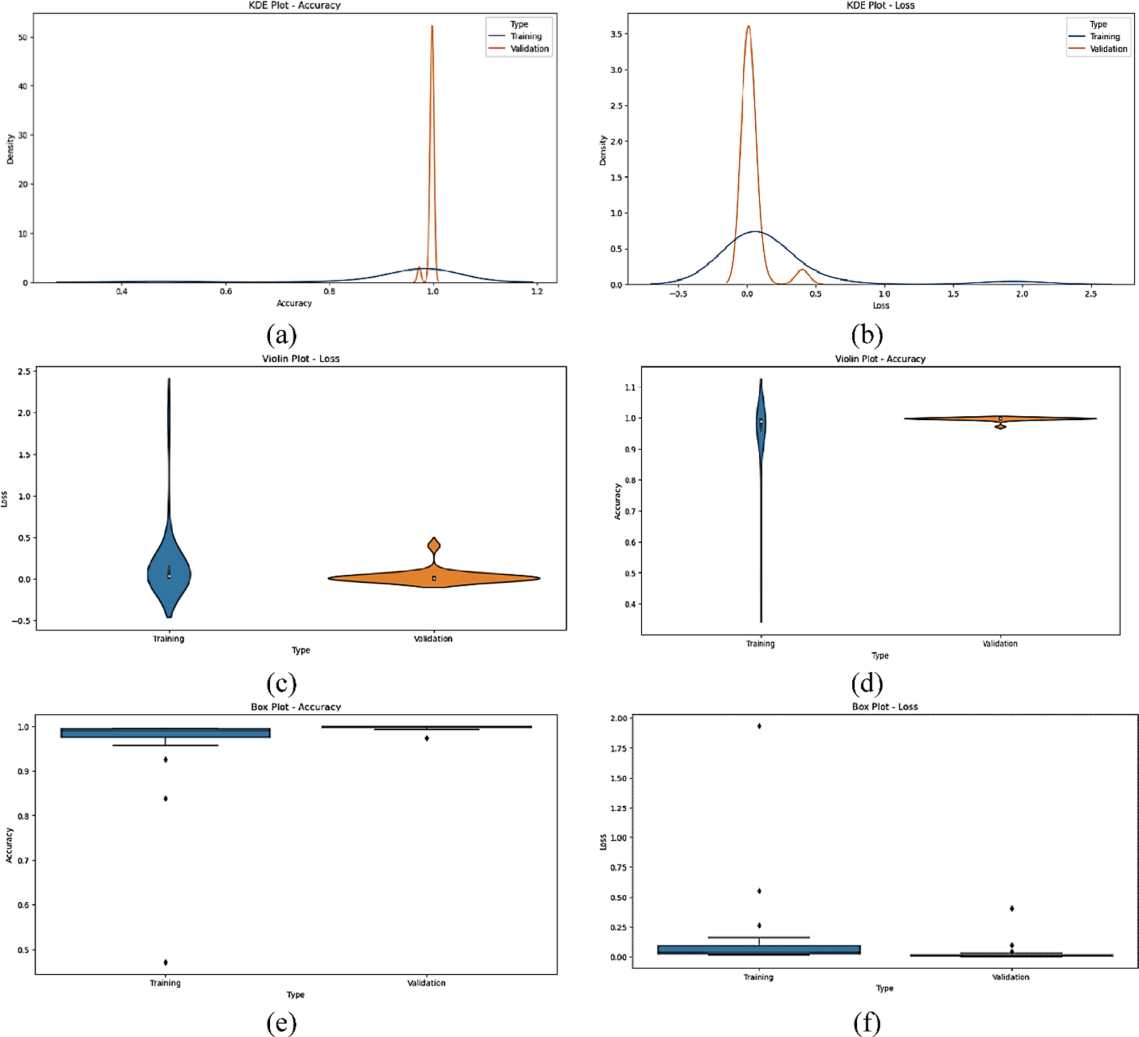
The statistical analysis of the proposed SNDA model for ASL Dataset. (**a**) KDE plot of accuracy, (**b**) KDE plot of loss, (**c**) Violin plot of loss, (**d**) Violin plot of accuracy, (**e**) Box plot of accuracy, and (**f**) Box plot of loss.

**Fig. 3:**
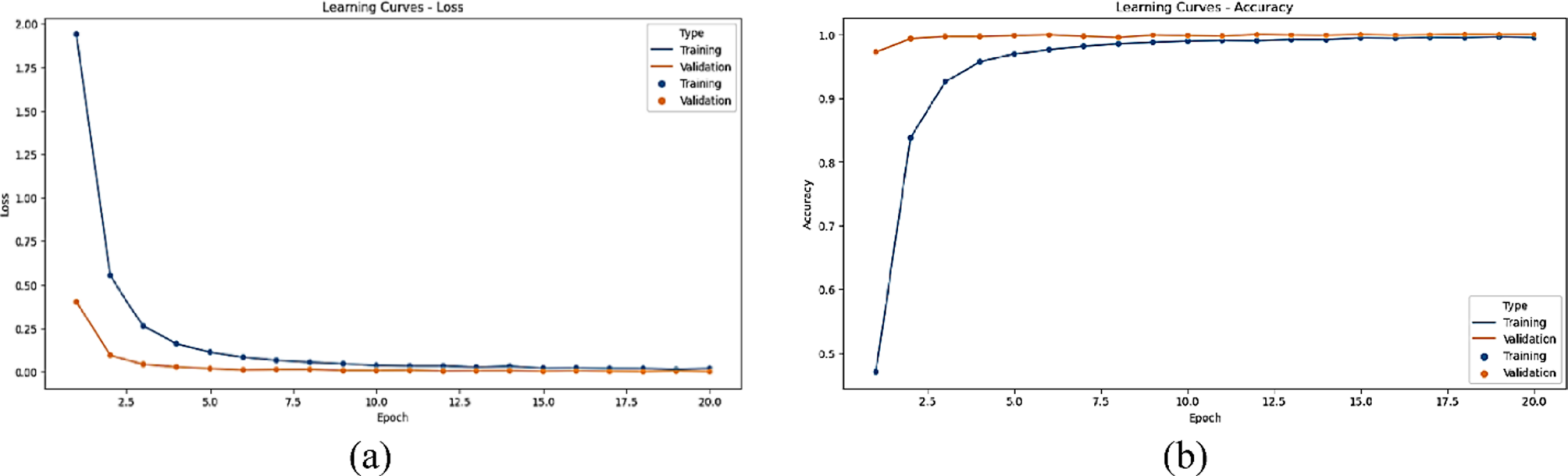
The learning curves of the proposed SNDA model for ASL Dataset. (**a**) Learning loss, (**b**) Learning accuracy.

**Fig. 4:**
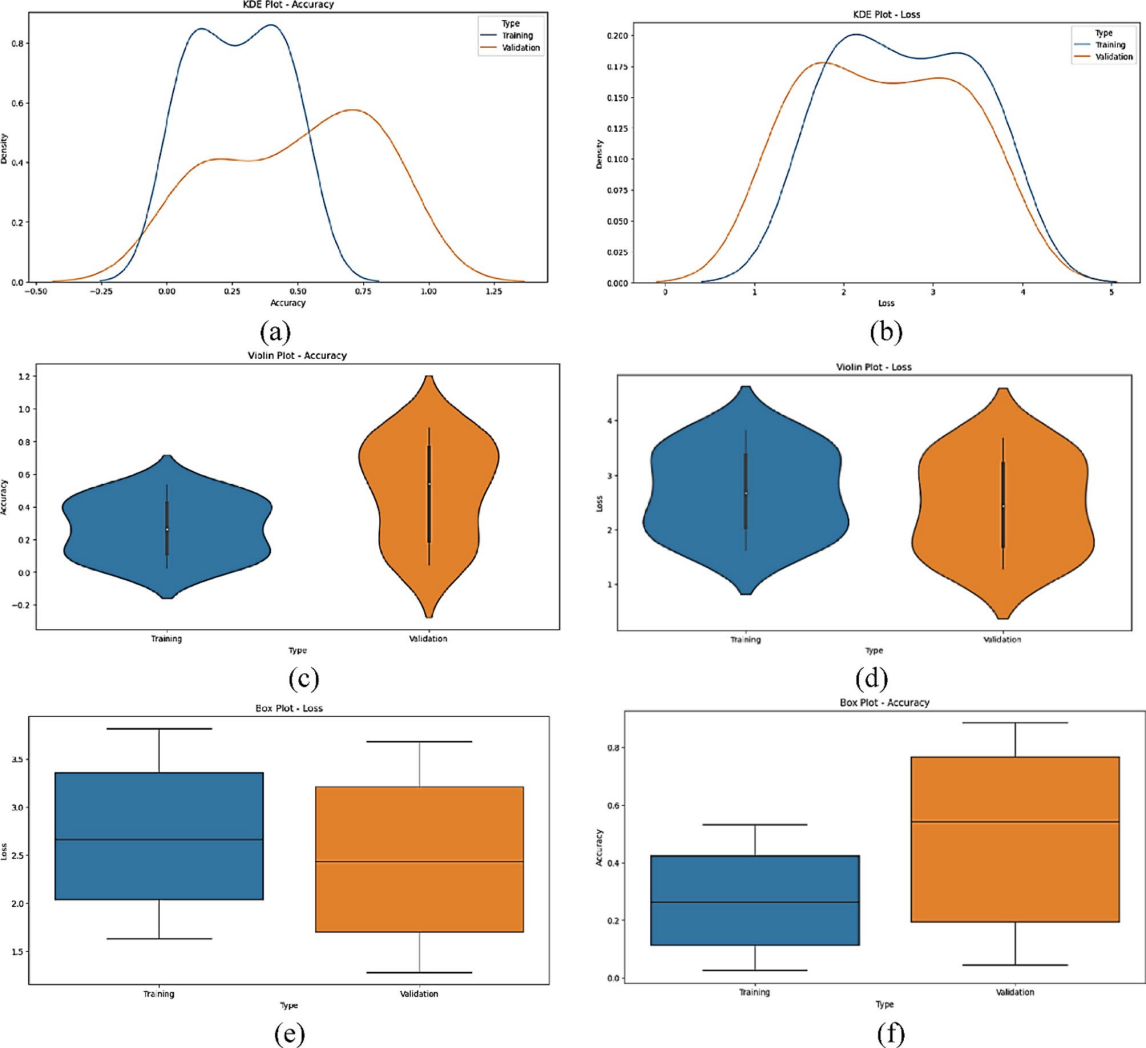
The statistical analysis of the proposed InceptionV3 with the Adam model for ASL Dataset. (**a**) KDE plot of accuracy, (**b**) KDE plot of loss, (**c**) Violin plot of accuracy, (**d**) Violin plot of loss, (**e**) Box plot of loss, and (**f**) Box plot of accuracy.

**Fig. 5:**
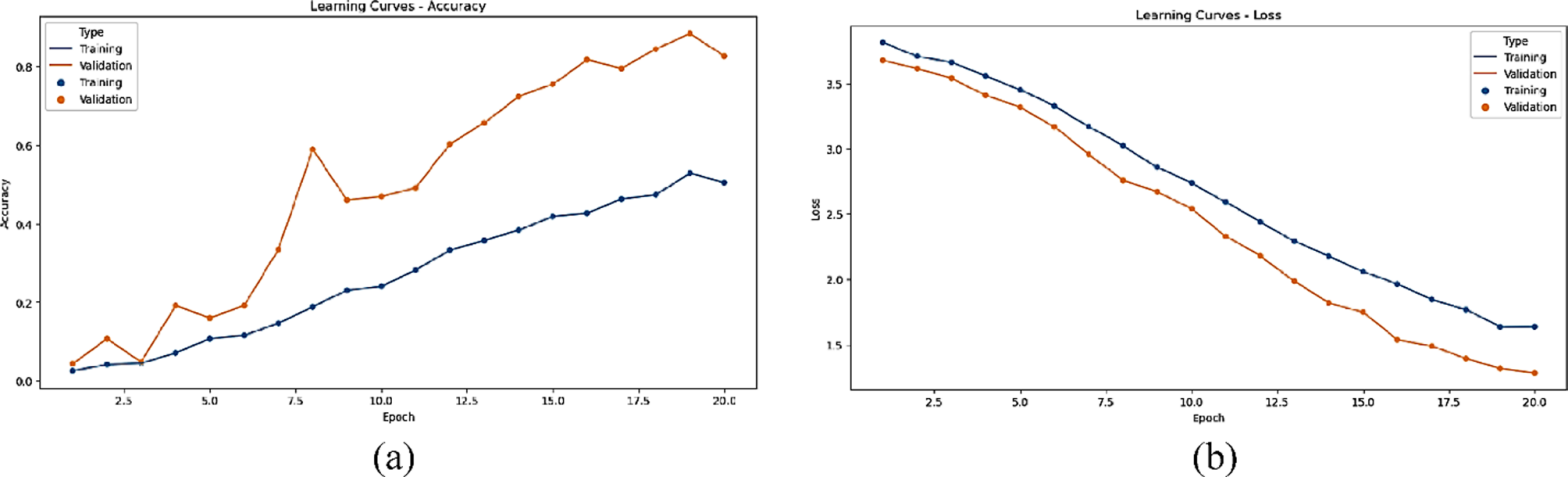
The learning curves of the proposed InceptionV3 with the Adam model. (**a**) Learning accuracy, (**b**) learning loss.

**Fig. 6:**
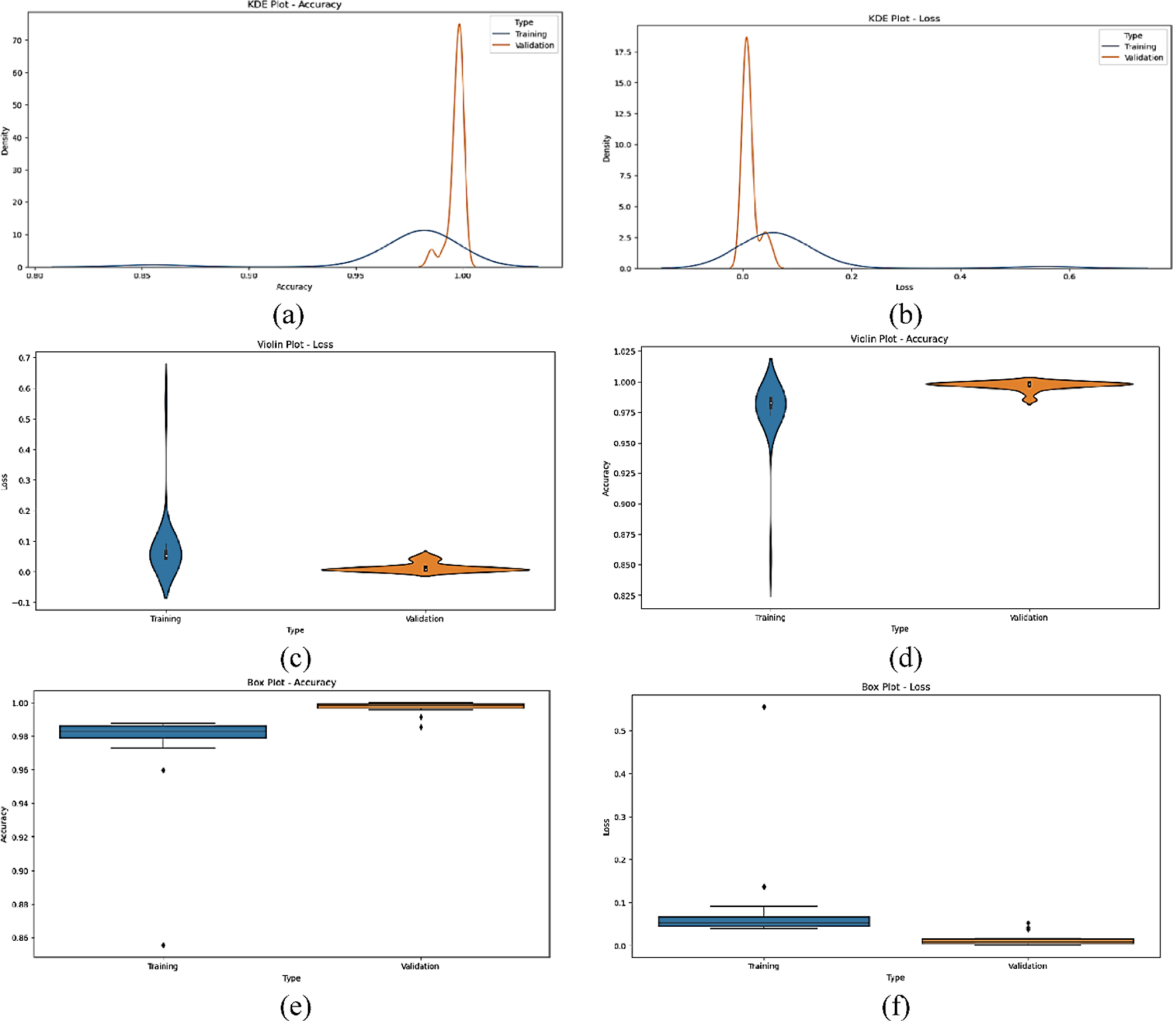
The statistical analysis of the proposed InceptionV3 with the Nadam model for ASL Dataset. (**a**) KDE plot of accuracy, (**b**) KDE plot of loss, (**c**) Violin plot of loss, (**d**) Violin plot of accuracy, (**e**) Box plot of accuracy, and (**f**) Box plot of loss.

**Fig. 7:**
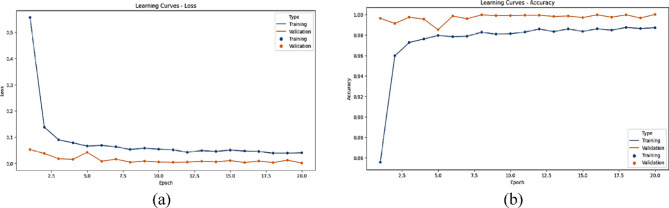
The learning curves of the proposed InceptionV3 with the Nadam model for ASL Dataset. (**a**) Learning loss, (**b**) Learning accuracy.

**Fig. 8:**
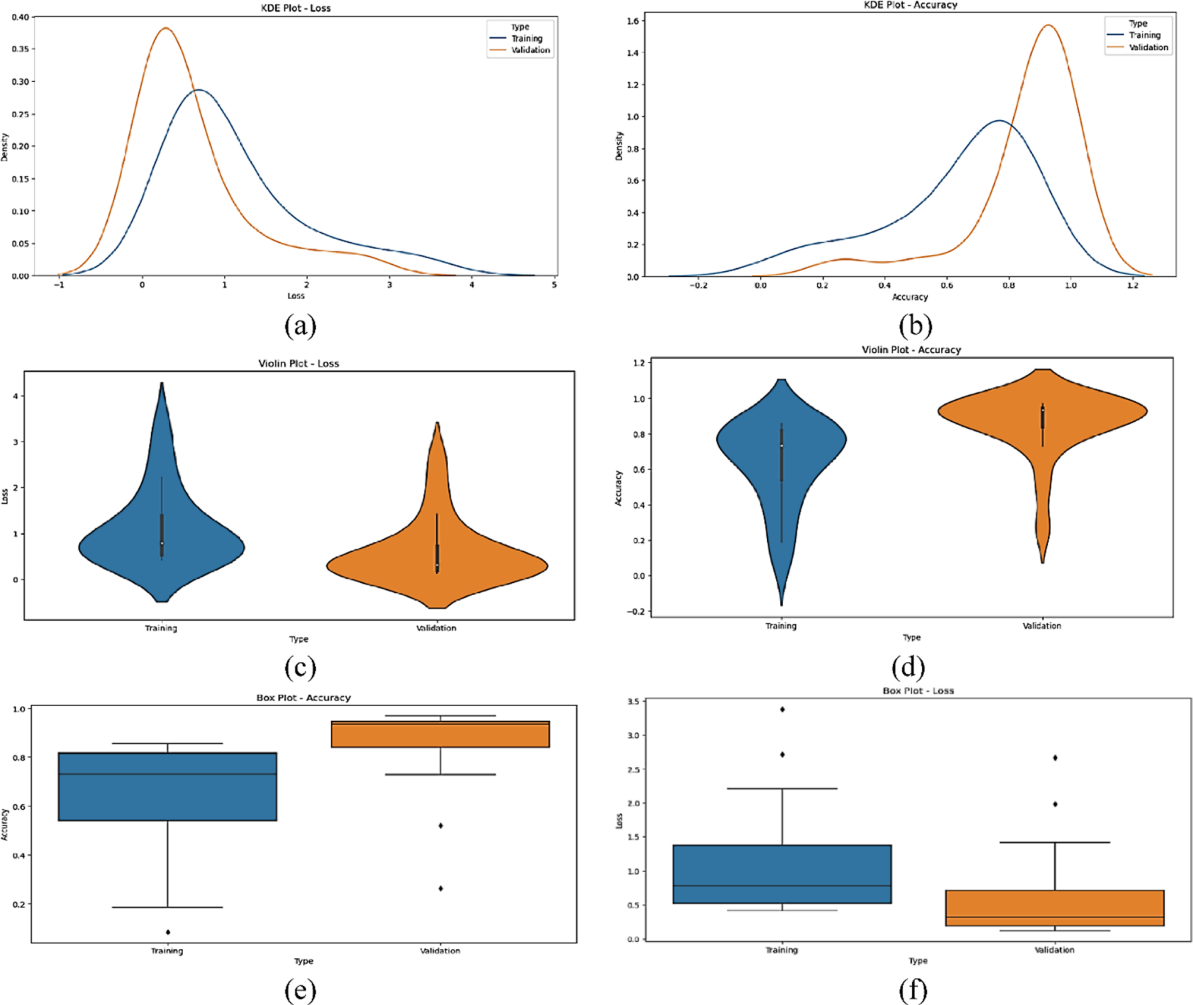
The statistical analysis of the proposed ResNet with the Nadam model for ASL Dataset. (**a**) KDE plot of loss, (**b**) KDE plot of accuracy, (**c**) Violin plot of loss, (**d**) Violin plot of accuracy, (**e**) Box plot of accuracy, and (f) Box plot of loss.

**Fig. 9:**
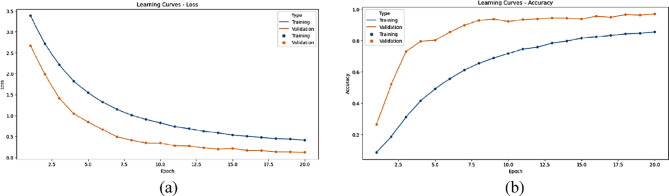
The learning curves of the proposed Resnet with the Nadam model for ASL Dataset. (**a**) Learning loss, (**b**) learning accuracy.

**Fig. 10:**
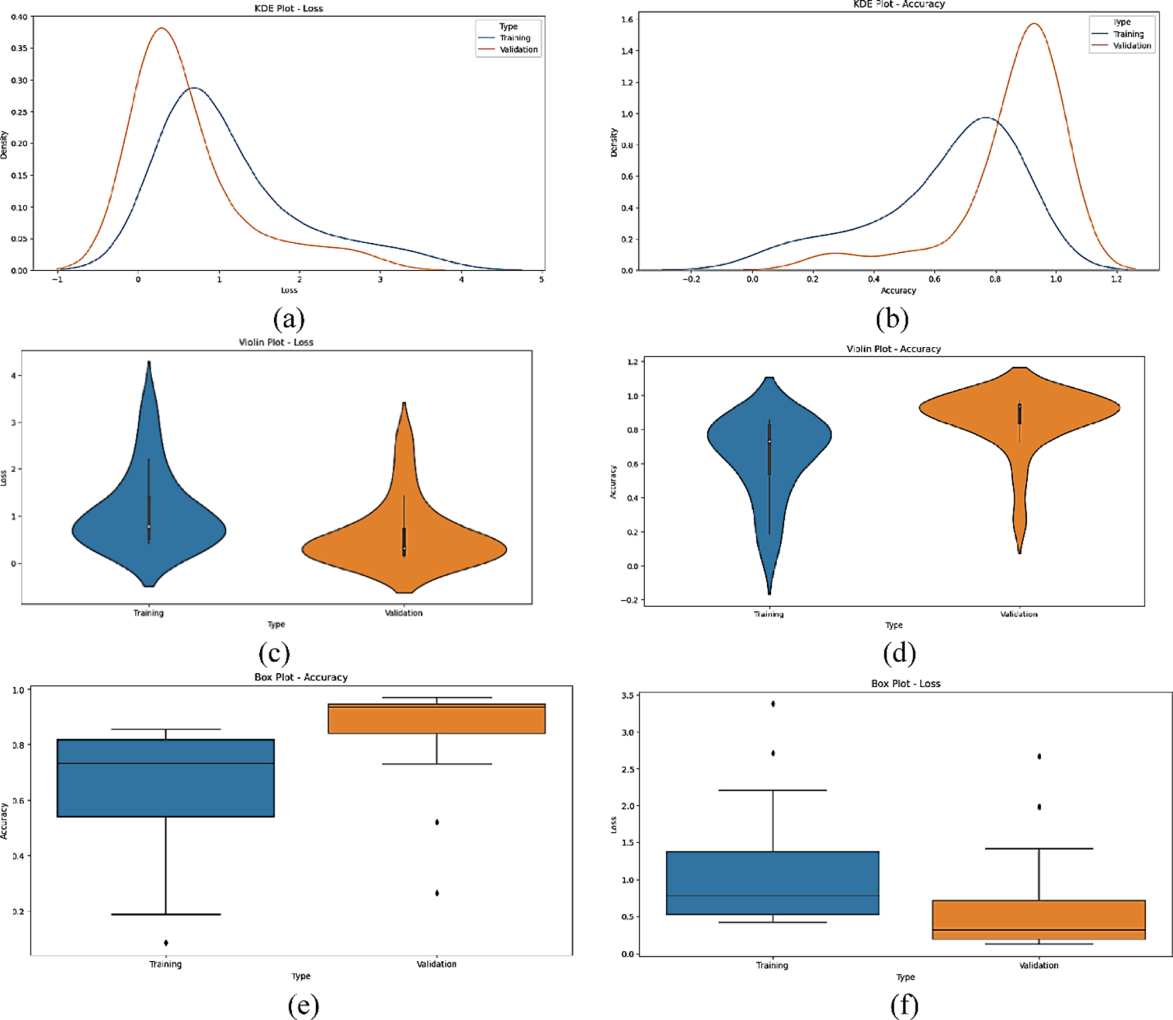
The statistical analysis of the proposed ResNet with the Adam model for ASL Dataset. (**a**) KDE plot of loss, (**b**) KDE plot of accuracy, (**c**) Violin plot of loss, (**d**) Violin plot of accuracy, (**e**) Box plot of accuracy, and (**f**) Box plot of loss.

**Fig. 11:**
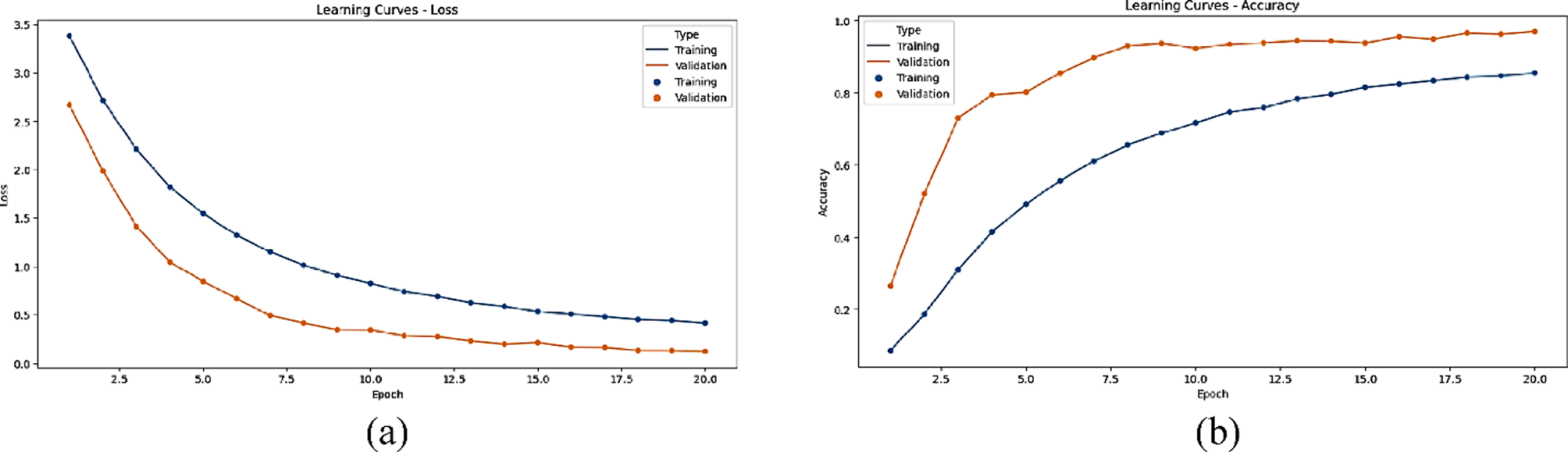
The learning curves of the proposed resnet with Adam model for ASL Dataset. (**a**) Learning loss, (**b**) learning accuracy.

The statistical performance of the proposed SNDA model is extensively analyzed in Fig. 2. This figure provides a comprehensive visualization using Kernel Density Estimation (KDE)^39^ plots to depict the distribution of accuracy and loss metrics during training and validation. Additionally, violin plots offer detailed insights into the spread and density of accuracy and loss values^40^. Furthermore, box plots carefully examine the quartile distribution, outliers, and median values of accuracy and loss for both training and validation. This multifaceted analysis provides a holistic understanding of the model’s characteristics and valuable insights for refinement. Figure 3 demonstrates the SNDA model’s learning curves for loss and accuracy during training. Figures 4 and 5 analyze the statistical performance and learning curves of the proposed InceptionV3 model^41^ with Adam optimization^42^ respectively. Figures 6 and 7 provide the same analysis for InceptionV3 with Nadam^6^ optimization. Figures 8 and 9 analyze the proposed ResNet model^36–38^ with Nadam optimization in terms of statistics and learning. Figures 10 and 11 assess the ResNet model using Adam optimization.

We provide a comprehensive comparative analysis of the proposed SNDA model against InceptionV3 and ResNet architectures using both Adam and Nadam optimizers, based on KDE plots, violin plots, box plots, and learning curves (Figs. 2, 3, 4, 5, 6, 7, 8, 9, 10 and 11). Across all visualizations, SNDA consistently demonstrates superior stability, lower variability, and more robust generalization, as indicated by its sharper KDE distributions, tighter violin and box plots, and smooth, well-behaved learning curves. In contrast, InceptionV3 and ResNet, particularly with Nadam, show broader distributions, increased variance, and signs of instability or overfitting, highlighting potential challenges in optimizer compatibility and model complexity. This detailed visual comparison underscores SNDA’s effectiveness and reliability for ASL classification tasks.

#### Experimental results for Sign-Language MNIST dataset

**Fig. 12:**
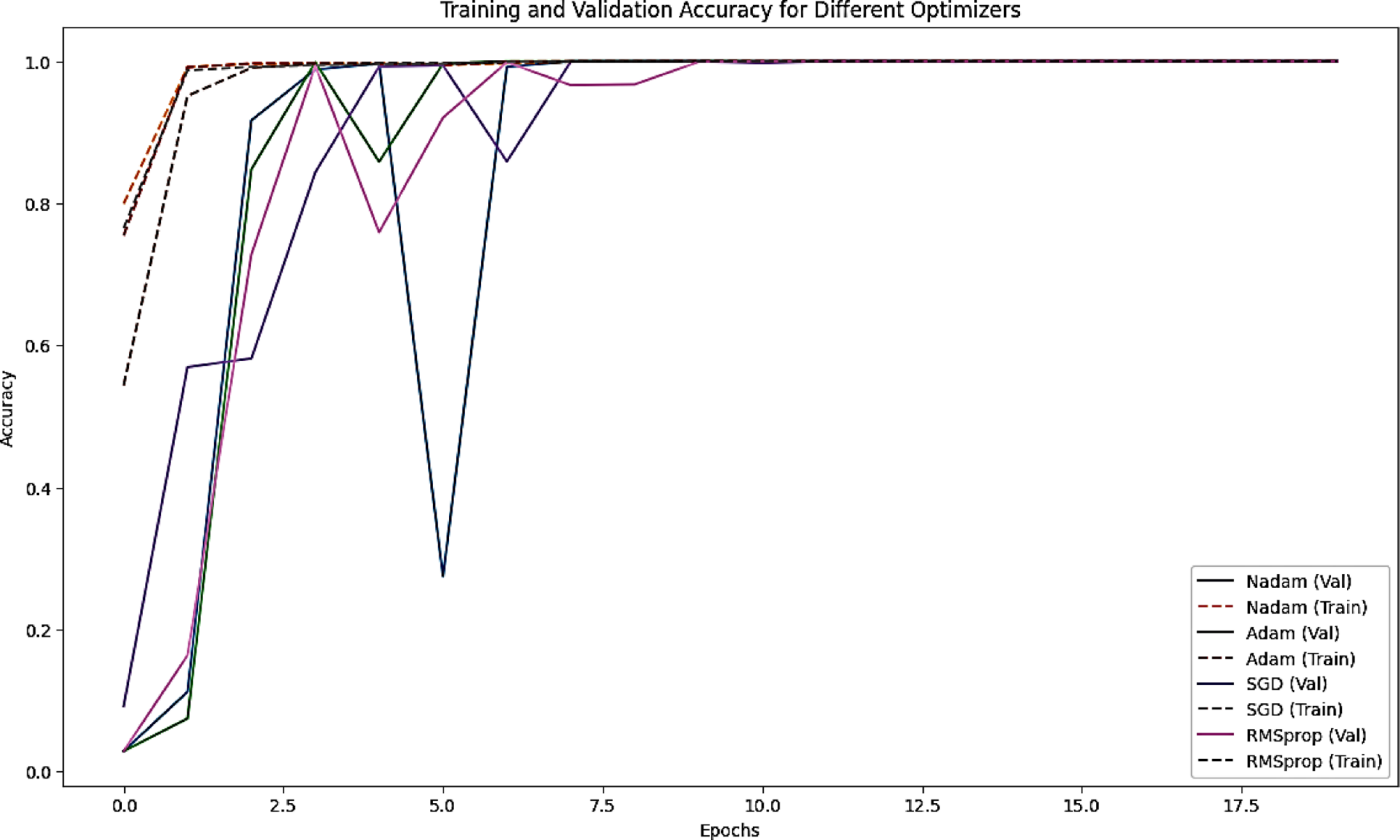
Training and validation accuracy curves for the model using different optimizers (Adam, Nadam, SGD, RMSprop) over epochs for the sign-language MNIST dataset.

In this section, we present the experimental results obtained from evaluating our proposed model on the Sign-Language MNIST dataset. The Sign-Language MNIST dataset is a collection of labeled images representing American Sign Language (ASL) letters, designed to facilitate research in sign language recognition. Our experiments aimed to assess the performance of the model in accurately classifying these sign language gestures. We conducted multiple runs to ensure the robustness of our results and compared the performance against a baseline model. The evaluation metrics, including accuracy, precision, recall, and F1-score, were used to provide a comprehensive analysis of the model’s effectiveness. The following subsections detail the experimental setup, the comparative analysis of the results, and the key insights derived from the evaluation. Figure 12 illustrates the performance comparison of each optimizer in terms of accuracy on both training and validation for the Sign-Language MNIST Dataset.

**Fig. 13:**
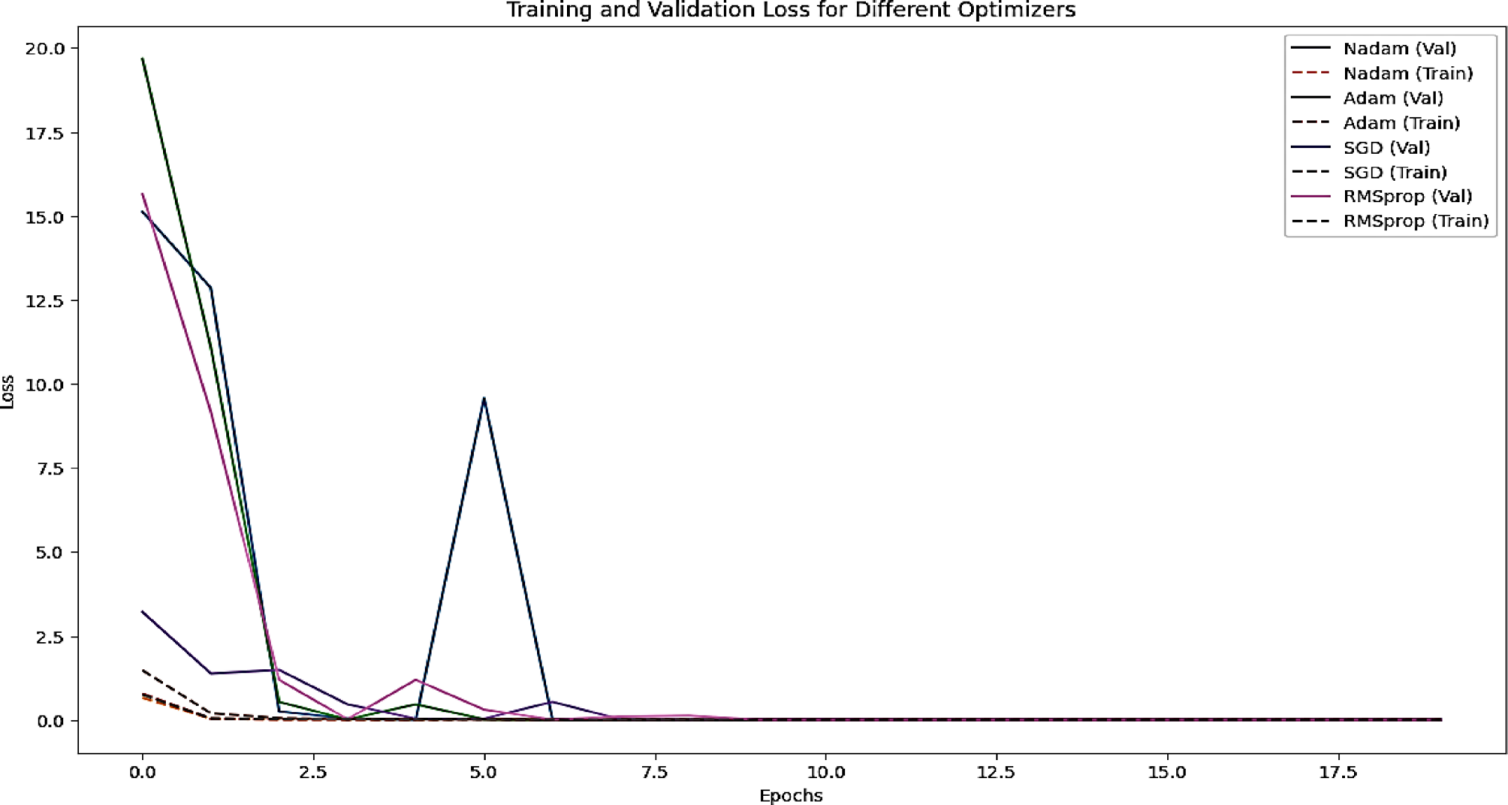
Training and validation loss curves for the model using different optimizers (Nadam, Adam, SGD, RMSprop) over epochs for the sign-language MNIST dataset.

**Fig. 14:**
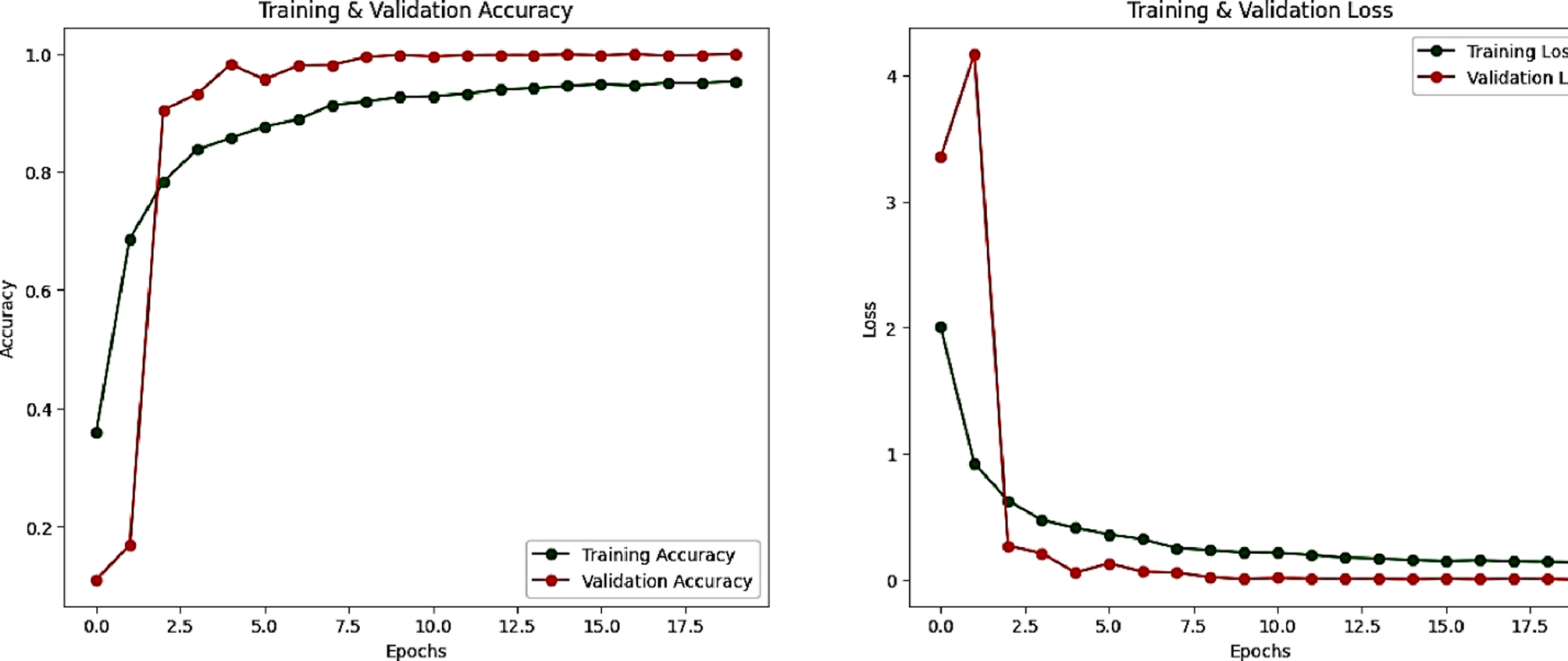
Training and validation accuracy and loss curves over epochs for both training and validation Sign-Language MNIST Dataset.

**Fig. 15:**
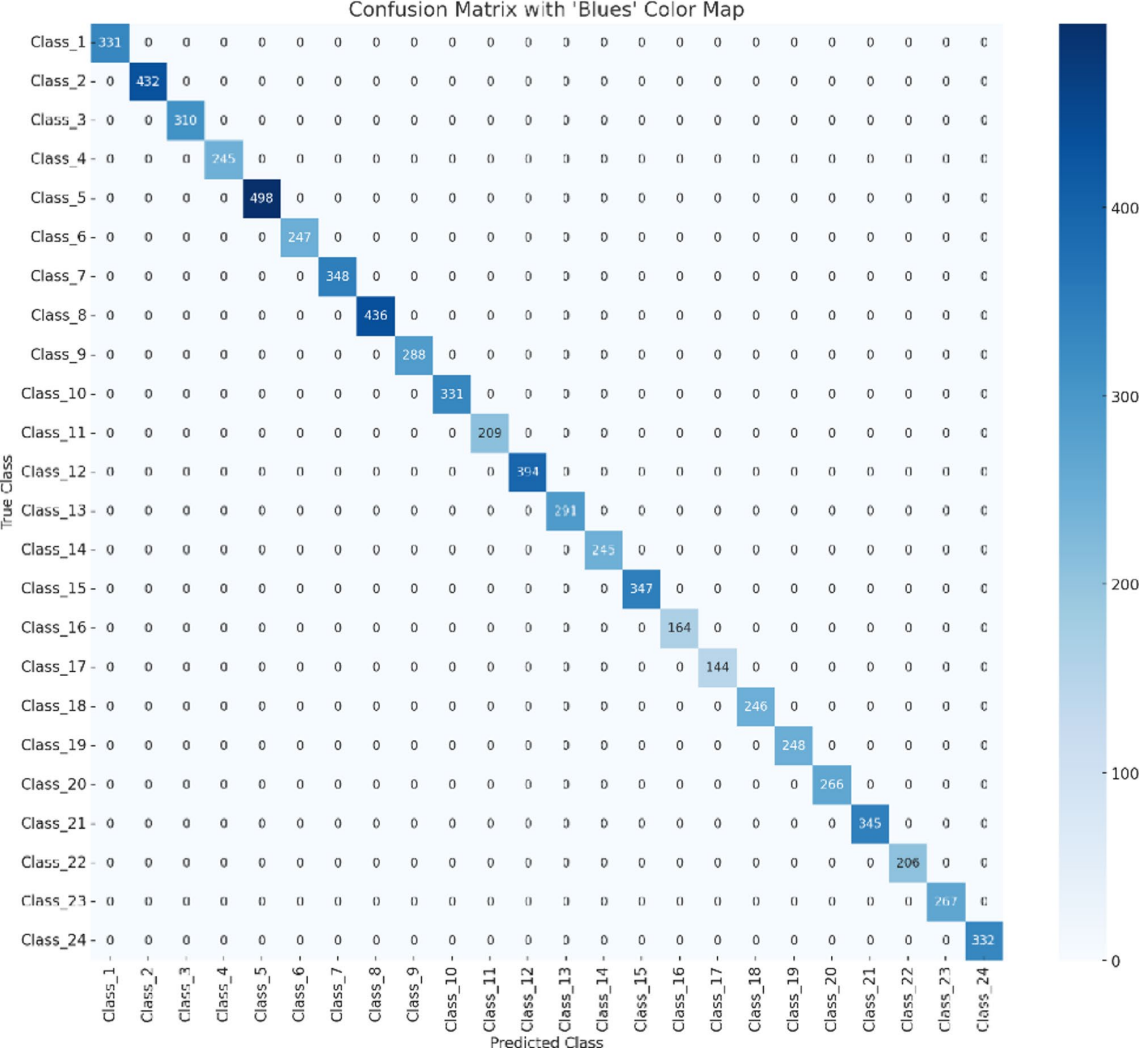
The confusion matrix for the sign-language MNIST dataset.

Figure 13 demonstrates the loss convergence behavior of each optimizer on both training and validation datasets and Figs. 14 and 15 for the learning curves and confusion matrix for the Sign-Language MNIST Dataset. Table 2 outlines the epochs at which the learning rate is reduced and the learning rate achieved for each optimizer, highlighting the dynamic adjustment strategy employed to optimize model convergence. Table 3 illustrates the Comparison of the SNDA Model and Baseline Model in terms of learning rate reduction schedule, learning rate, test accuracy, and paired t-test results.

**Table 2:** Learning rate reduction schedule for different optimizers (Nadam, adam, SGD, RMSprop) during training.

Optimizer	Learning rate reduction epochs	Final learning rate
Nadam	Epoch 7: 0.0005 Epoch 10: 0.00025 Epoch 12: 0.000125 Epoch 14: 6.25e-05 Epoch 16: 3.125e-05 Epoch 18: 1.5625e-05 Epoch 20: 1e-05	1e-05
Adam	Epoch 6: 0.0005 Epoch 9: 0.00025 Epoch 11: 0.000125 Epoch 13: 6.25e-05 Epoch 15: 3.125e-05 Epoch 17: 1.5625e-05 Epoch 19: 1e-05	1e-05
SGD	Epoch 12: 0.005 Epoch 14: 0.0025 Epoch 16: 0.00125 Epoch 18: 0.000625 Epoch 20: 0.0003125	0.0003125
RMSprop	Epoch 6: 0.0005 Epoch 9: 0.00025 Epoch 13: 0.000125 Epoch 15: 6.25e-05 Epoch 17: 3.125e-05 Epoch 19: 1.5625e-05	1.5625e-05

Figure 15 demonstrates strong classification performance, with all classes correctly identified and no visible misclassifications. However, we acknowledge that Class_11 and Class_16 exhibit lower correct predictions, potentially indicating difficulty due to class complexity or similarity with other gestures.

The results indicate that the model is highly effective on the given dataset, achieving perfect accuracy with all optimizers. However, the learning rate reduction schedules and learning rates vary across optimizers, reflecting differences in their convergence behavior:

- Nadam and Adam converge faster, requiring earlier learning rate adjustments.
- SGD converges more slowly, requiring fewer but larger learning rate reductions.
- RMSprop behaves similarly to Adam but with slightly fewer reductions.
- While all optimizers achieve perfect accuracy, the choice of optimizer may still matter in terms of training efficiency and robustness to hyperparameters.

**Fig. 16:**
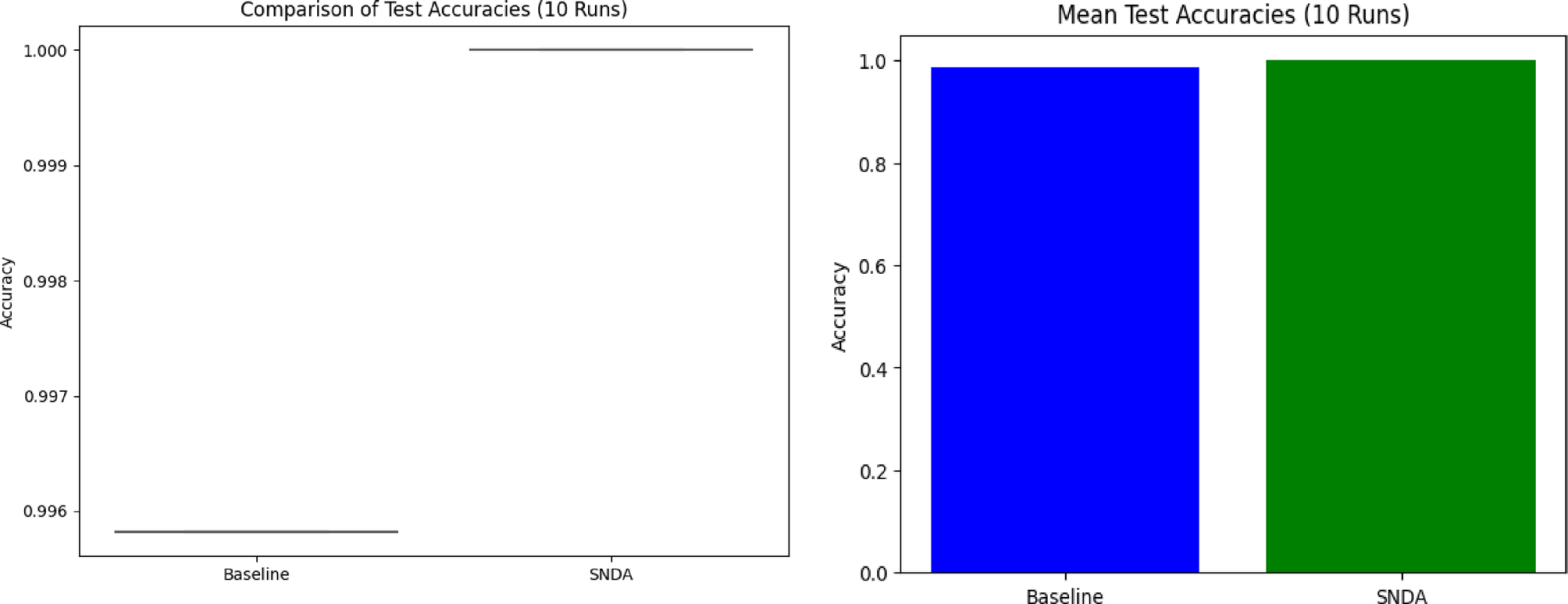
Comparison of test accuracies over 10 runs between the baseline model and the SNDA model.

Figure 16 highlights the consistent performance and superior accuracy of the SNDA model compared to the baseline across multiple runs.

**Table 3:** Comparison of the SNDA model and baseline model in terms of learning rate reduction schedule, learning rate, test accuracy, and paired t-test results.

Model	Learning rate reduction epochs	Final learning rate	Test accuracy	Paired t-test results
SNDA model	Epoch 7: 0.0005 Epoch 12: 0.00025 Epoch 14: 0.000125 Epoch 16: 6.25e-05 Epoch 18: 3.125e-05 Epoch 20: 1.5625e-05	1.5625e-05	100.00%	t = inf, p = 0.0000
Baseline model	-	-	99.58%	-

i) Learning rate reduction:

- The SNDA model undergoes multiple learning rate reductions during training, starting from Epoch 7 and continuing until Epoch 20. The learning rate for the SNDA model is 1.5625e-05.

ii) Test accuracy:

- The SNDA model achieves 100.00% test accuracy, indicating perfect performance on the test set. The Baseline model achieves 99.58% test accuracy, which is slightly lower than the SNDA model.

iii) Statistical significance testing:

- The paired t-test results show a t-statistic of infinity (inf) and a p-value of 0.0000, indicating that the difference in accuracy between the SNDA and Baseline models is statistically significant (*p* < 0.05).

#### Evaluation results and comparative analysis

Table 4 provides an overview of the performance metrics for different ASL recognition models. These models have been evaluated based on their accuracy, sensitivity, specificity, and precision. The table includes four established models, namely InceptionV3 + Adam, Inception V3 + Nadam, Resnet + Nadam, and Resnet + Adam, along with the proposed architecture called Sign Nevestro Densenet Attention (SNDA). Accuracy represents the correctness of the model’s predictions, while sensitivity measures the model’s ability to correctly identify positive instances. Specificity, on the other hand, assesses the model’s accuracy in identifying negative instances. Precision quantifies the proportion of true positive predictions out of all positive predictions made by the model.

**Table 4:** The evaluation results of the proposed inceptionV3 + Adam, inception V3 + Nadam, Resnet + Nadam, Resnet + Adam, and SNDA models for ASL dataset.

Model architecture	Accuracy (%)	Sensitivity (%)	Specificity (%)	Precision (%)	Training time (s)	Testing time (s)	Estimated parameters (Millions)	Per-image inference time	Total inference time (8000 images)
InceptionV3 + Adam	99.04	99.01	99.07	99.07	1850	**45**	~ 23.9 M	~ 12–15 ms (GPU)	~ 90–120 s (GPU)
InceptionV3 + Nadam	99.96	99.50	99.53	99.50	1920	**48**	~ 23.9 M	~ 12–15 ms (GPU)	~ 90–120 s (GPU)
ResNet50 + Nadam	97.25	97.56	98.13	98.04	1420	**35**	~ 25.6 M	~ 10–13 ms (GPU)	~ 80–100 s (GPU)
ResNet50 + Adam	96.94	97.09	96.80	96.62	1380	**34**	~ 25.6 M	~ 10–13 ms (GPU)	~ 80–100 s (GPU)
Proposed SNDA Model	**99.76**	**100.00**	**99.53**	**99.50**	**980**	**25**	**~ 11 M (8 M + 3M)**	**7–10 ms (GPU)**	**60–80 s (GPU)**

To provide a clearer assessment of the model’s practical utility, we highlight the relationship between sensitivity, specificity, and false positive rates (FPR) in our evaluation. From the reported results, the SNDA model achieves 100% sensitivity, meaning it correctly identifies all positive ASL gestures without missing any. However, specificity is 99.53%, indicating a minimal false positive rate (FPR = 1 - Specificity = 0.47%). This balance suggests that while SNDA excels at recognizing gestures correctly, occasional misclassifications of non-ASL inputs or different gestures may occur. When compared to other models:

- InceptionV3 + Nadam achieves 99.50% sensitivity and 99.53% specificity, resulting in a comparable FPR of 0.47%.
- ResNet + Nadam and ResNet + Adam show lower sensitivity and specificity, leading to higher FPRs (1.87% and 3.20%, respectively).

These results confirm that SNDA maintains a superior balance between high sensitivity and specificity while minimizing false positives, reinforcing its reliability for ASL recognition tasks.

**Fig. 17:**
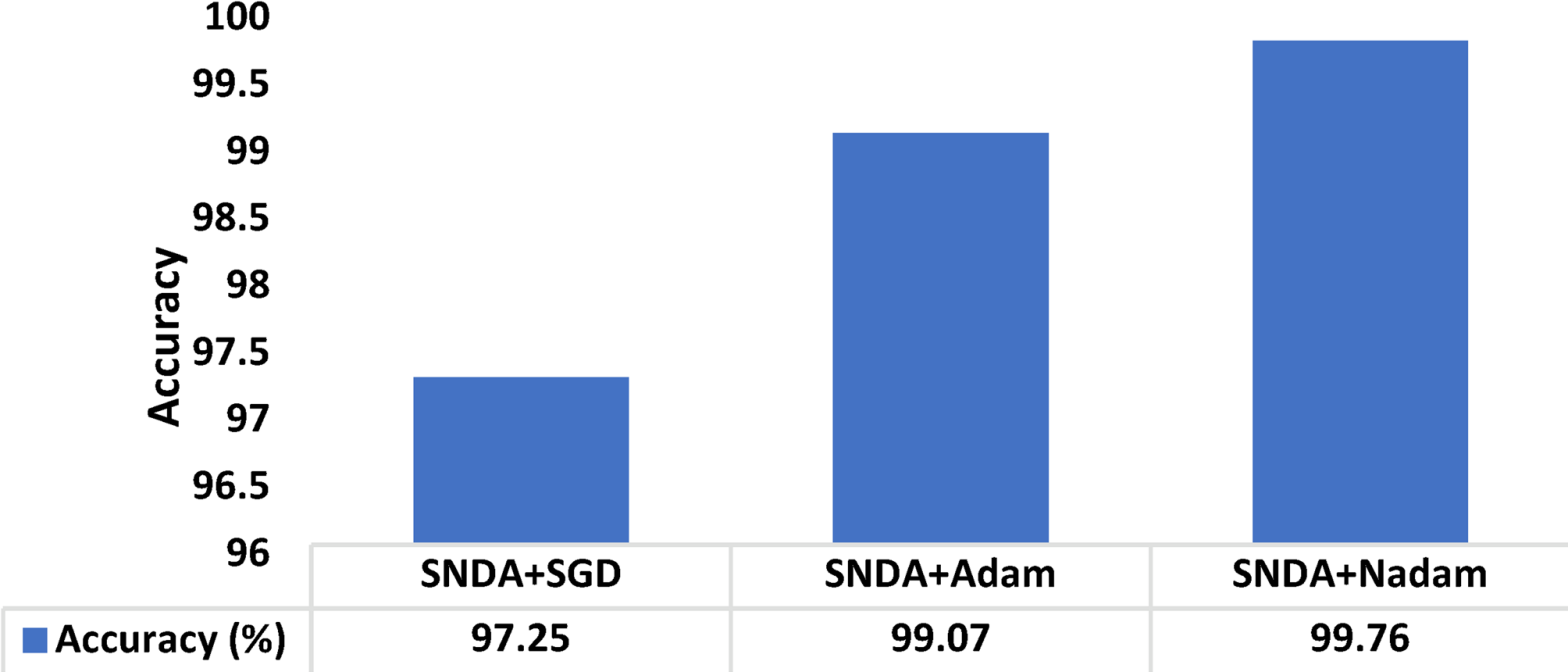
The comparison between the SNDA with SGD, Adam, and Nadam optimizers in terms of accuracy.

The choice of the Nadam optimizer was based on its adaptive learning rate and momentum-based approach, which have been shown to enhance convergence speed and stability. While our experimental results demonstrated efficient training and high accuracy, we compare the Nadam optimizer with Adam and SGD for the proposed SNDA model in terms of the accuracy shown in Fig. 17.

**Fig. 18:**
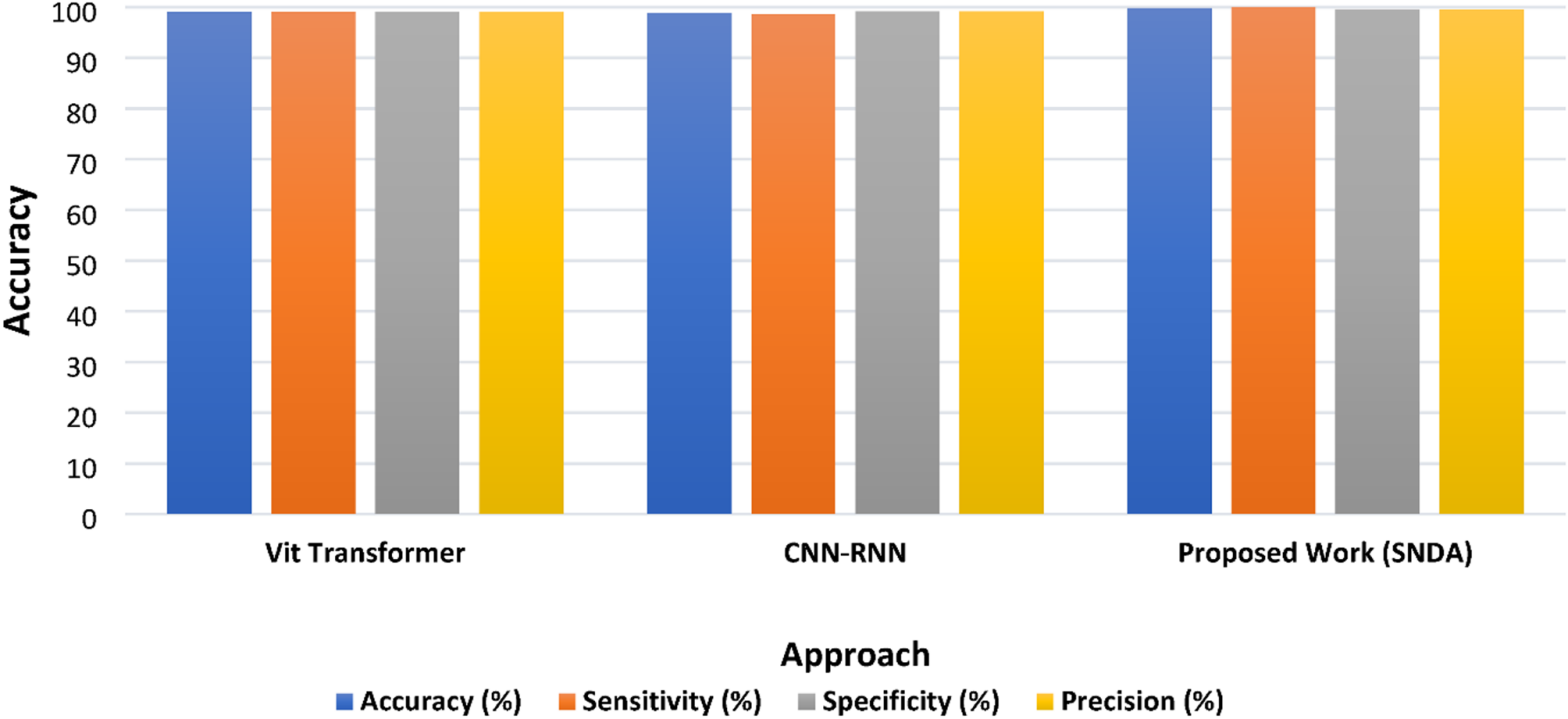
The comparative study between the Vit transformer, CNN-RNN, and the proposed SNDA for the ASL dataset.

The selection of DenseNet for SNDA was based on its proven efficiency in feature propagation and reduced parameter redundancy, making it well-suited for ASL recognition, where fine-grained spatial features are crucial. We acknowledge the importance of comparing SNDA with other attention-based models such as Transformers and hybrid CNN-RNN architectures. While our primary focus was to enhance CNN-based recognition with attention mechanisms, we expanded our analysis to include the Vit transformer and the CNN-RNN as shown in Fig. 18.

The number of samples (support) for each class varies, with the largest class (Class 4) having 498 samples and the smallest class (Class 17) having 144 samples. Despite the varying class sizes, the model achieves perfect performance across all classes, demonstrating its robustness and ability to handle class imbalance. Implications: The results suggest that the model is highly accurate and generalizes well to all classes in the dataset. The perfect scores indicate that the model is capable of correctly classifying every sample in the test set, with no false positives or false negatives.

Among the established models, Resnet + Nadam achieves an accuracy of 97.25%, sensitivity of 97.56%, specificity of 98.13%, and precision of 98.04%. Resnet + Adam follows closely with an accuracy of 96.94%, sensitivity of 97.09%, specificity of 96.80%, and precision of 96.62%. Inception V3 + Nadam exhibits remarkable performance with an accuracy of 99.96%, a sensitivity of 99.50%, a specificity of 99.53%, and a precision of 99.50%. Similarly, Inception V3 + Adam demonstrates high accuracy, sensitivity, specificity, and precision, with values of 99.04%, 99.01%, 99.07%, and 99.07%, respectively.

The proposed architecture, SNDA, showcases exceptional performance in ASL recognition. It achieves an accuracy of 99.76%, perfect sensitivity of 100.00%, specificity of 99.53%, and precision of 99.50%. These results highlight the effectiveness of SNDA in accurately recognizing ASL gestures and its potential to contribute to bridging communication gaps and fostering inclusivity for the Deaf and hard-of-hearing community.

### Complexity, inference, and training time

The computational complexity of the proposed SNDA model is influenced by the dataset scale and architectural depth. The American Sign Language (ASL) Recognition Dataset used in this work consists of 56,000 low-resolution training images and 8,000 test images, each of size 64 × 64 × 3. The SNDA model integrates a DenseNet121 backbone, pre-trained on ImageNet, with additional self-attention and fully connected layers, resulting in an estimated 11 million parameters—approximately 8 M from DenseNet121 and 3 M from the added custom layers. From a theoretical perspective, the total computational complexity can be approximated as O (n × m), where n denotes the number of input samples and m represents the number of trainable parameters. The reduced input resolution significantly lowers floating-point operations (FLOPs) and memory usage per image compared to standard input sizes (224 × 224), contributing to improved training efficiency and deployment feasibility. Empirically, inference time for a single image is approximately 7–10 milliseconds on a GPU (NVIDIA RTX 3060), 70–100 milliseconds on a CPU (Intel i7), and 200–300 milliseconds on an edge device (Raspberry Pi 4). For the entire test set of 8,000 images, the total inference time is ~ 60–80 s on GPU, assuming batched prediction with minimal I/O overhead. Training time, using a batch size of 32 over 50 epochs, is approximately 2.5 h on a single GPU, 10–12 h on a CPU, and under 1.5 h on a TPU, with efficiency enhanced by the Nadam optimizer and pre-trained DenseNet feature extraction that illustrated on Table 4. The O(n*m) relationship reflects the linear scaling of computational demand with both dataset size and model complexity, underscoring the importance of optimizing both when designing models for real-time ASL recognition.

### Discussion

American Sign Language (ASL) plays a crucial role as a communication tool for the deaf and hard-of-hearing community. Recent advancements in computer vision and machine learning have led to the development of various ASL recognition techniques. However, there is a need for further improvement in accuracy and robustness. This study presents a comprehensive comparison of different ASL recognition techniques and introduces a novel architecture called Sign Nevestro Densenet Attention (SNDA). The performance of all methods is evaluated on the ASL Recognition Dataset, ensuring a representative evaluation.

The experimental results demonstrate the superior performance of SNDA compared to other methods in terms of accuracy, sensitivity, specificity, and precision. SNDA achieves an accuracy of 99.76%, perfect sensitivity, and high specificity and precision. These results validate the effectiveness of the proposed architecture in ASL gesture recognition and highlight its potential to bridge communication gaps and foster inclusivity for the Deaf and hard-of-hearing community.

Despite breakthroughs in computer vision and machine learning, current ASL recognition techniques lag in accuracy and robustness. This hinders effective communication for the deaf and hard-of-hearing community, limiting accessibility and social interaction.

Misinterpreting ASL gestures is a significant challenge faced by current ASL recognition techniques. Imperfect accuracy can lead to misunderstandings and communication breakdowns, which can be frustrating and alienating for the Deaf and hard-of-hearing community. These limitations highlight the need for more accurate recognition systems that can minimize misinterpretations and improve communication accessibility.

Sensitivity to variations is another critical issue in ASL recognition. Lighting conditions, camera angles, and individual signing styles can introduce variations that confuse current models. ASL recognition systems must be able to accurately identify gestures regardless of these variations to ensure reliable and effective communication. While many existing methods perform well in controlled lab settings, practical applications such as ASL translation systems and assistive technologies require robustness beyond these controlled environments.

To address these challenges, this study proposes Sign Nevestro Densenet Attention (SNDA), a novel architecture specifically designed for ASL recognition. SNDA aims to significantly improve recognition accuracy^44,45^. By achieving these goals, SNDA has the potential to revolutionize computer vision for ASL recognition, fostering inclusivity and accessibility for the Deaf and hard-of-hearing community.

The presented experimental results demonstrate the effectiveness of SNDA in achieving these goals. SNDA outperforms other methods, including Resnet + Nadam, Resnet + Adam, Inception + Nadam, and Inception + Adam, in terms of accuracy, sensitivity, specificity, and precision. The high accuracy, perfect sensitivity, and high specificity and precision of SNDA validate its potential for accurate ASL gesture recognition. These results support the claim that SNDA can minimize misinterpretations and communication barriers, accurately identify gestures under varying conditions, and function effectively in practical applications.

The findings of this study contribute to the ongoing advancement of ASL recognition technology. By introducing SNDA, a novel architecture that combines attention mechanisms with established architectures, this research demonstrates the potential for enhancing deep learning models in specific application domains. The superior performance of SNDA compared to other methods sets new standards for accuracy and robustness in ASL recognition. The ASL Recognition Dataset provides a substantial collection of labeled ASL gestures, making it a valuable resource for training and evaluating recognition models. While the dataset includes diverse hand gestures and linguistic expressions^46^.

## Conclusion and future work

This study focused on the important task of American Sign Language (ASL) recognition, which plays a crucial role as a communication tool for the deaf and hard-of-hearing community. While advancements in computer vision and machine learning have contributed to ASL recognition techniques, there is still a need for further improvement in accuracy and robustness to enhance communication accessibility. To address this need, we introduced a novel architecture called Sign Nevestro Densenet Attention (SNDA) and conducted a comprehensive comparison of different ASL recognition techniques. SNDA utilizes the Nadam optimizer, which enables faster convergence and stable optimization during model training. The experimental results demonstrated the superior performance of SNDA compared to other methods in terms of accuracy, sensitivity, specificity, and precision. SNDA achieved an impressive accuracy of 99.76%, perfect sensitivity, and high specificity and precision. These results validate the effectiveness of the proposed architecture in ASL gesture recognition and highlight its potential to bridge communication gaps and foster inclusivity for the Deaf and hard-of-hearing community. The introduction of SNDA in this research contributes to the ongoing advancement of ASL recognition technology, setting new standards for accuracy and robustness. By utilizing attention mechanisms in conjunction with established architectures, our work demonstrates the potential for enhancing deep learning models in specific application domains.

While this study has advanced ASL recognition significantly, there remain avenues for further research and improvement. Potential future directions include expanding datasets to encompass a broader range of gestures and styles for more comprehensive evaluation, exploring multi-modal approaches integrating depth and audio cues for enhanced accuracy, investigating transfer learning techniques from large-scale datasets to improve performance for dynamic environments, integrating user feedback mechanisms for ongoing model refinement to gauge performance, usability, and impact across various applications such as assistive technologies and educational tools.

## Data Availability

Data availability statement: The dataset used in this study is public and all test data are available at: https://www.kaggle.com/datasets/rahulmakwana/american-sign-language-recognition.

## Code availability

The custom code and algorithms used to generate the results in this study are publicly available under an open-source license at: https://github.com/tarekhemdan/ASL. A versioned DOI (10.5281/zenodo.15725210) has been minted via Zenodo to ensure long-term accessibility. All test data required to replicate the findings are included in the repository (https://zenodo.org/records/15725210).
